# Effects of Sleeve Gastrectomy on Fecal Gut Microbiota and Short-Chain Fatty Acid Content in a Rat Model of Polycystic Ovary Syndrome

**DOI:** 10.3389/fendo.2021.747888

**Published:** 2021-11-11

**Authors:** Wei Lin, Lingying Wen, Junping Wen, Guangda Xiang

**Affiliations:** ^1^ The First School of Clinical Medicine, Southern Medical University, Guangzhou, China; ^2^ Department of Endocrinology, General Hospital of Central Theater Command, Wuhan, China; ^3^ Department of Endocrinology, Shengli Clinical Medical College of Fujian Medical University, Fujian Provincial Hospital, Fuzhou, China; ^4^ Department of Neonatology, The First Affiliated Hospital of Fujian Medical University, Longyan, China

**Keywords:** sleeve gastrectomy, gut microbiota, short-chain fatty acids, polycystic ovary syndrome, rat

## Abstract

**Purpose:**

Sleeve gastrectomy (SG) is a surgical intervention for polycystic ovary syndrome (PCOS), especially for patients with obesity. Here, we explored the effects of SG on the gut microbiota of rats with PCOS and investigated the association between the intestinal flora and efficacy of SG in PCOS.

**Methods:**

Dehydroepiandrosterone (DHEA) injection was administered alone and in combination with a high-fat diet to induce PCOS in rats. SG was performed in rats with PCOS, and the effects of SG on the fecal and gut microbiota and the short-chain fatty acid (SCFA) content were observed. Furthermore, the association among gut microbiota, SCFA content and hyperandrogenism or other hallmarks of PCOS was evaluated.

**Results:**

The abundance of Firmicutes reduced and that of Bacteroidetes increased in response to SG in the DHEA-induced PCOS rat model. At the genus level, the abundances of *Bacteroides* and *Blautia* increased and those of *Ruminococcus*, *Clostridium*, and *Alistipes* reduced distinctly in the PCOS-SG groups. Moreover, the levels of fecal SCFAs, especially butyric acid, reduced after SG. SG significantly ameliorated PCOS-related symptoms such as hyperandrogenism, disrupted ovary function, and impaired glucose tolerance. *Bacteroides* and *Blautia* exhibited a negative correlation and *Ruminococcus*, *Clostridium*, and *Alistipes* exhibited a positive correlation with the levels of fecal SCFAs, luteinizing hormone, testosterone, and inflammatory factors.

**Conclusions:**

The amelioration of PCOS-related reproductive and metabolic disorders following SG was associated with the regulation of microbial taxa and SCFA content. Our findings provide a novel perspective on the microbial mechanisms in PCOS after SG.

## Introduction

Polycystic ovary syndrome (PCOS) is a heterogeneous condition characterized by hyperandrogenism, oligo/anovulation, and polycystic ovaries (1). It is one of the major causes of metabolic disorders in women of reproductive age. More than 50% of the women with PCOS are either overweight or have obesity (2). To date, the exact pathogenesis of PCOS remains uncharacterized. Given the limitations of the interventions currently used for treating PCOS-associated metabolic disorders, novel treatment strategies are warranted.

While promising, only a few studies have shown that bariatric surgery may be beneficial for individuals with PCOS (3, 4). Multiple studies have suggested that significant and persistent weight loss is an important outcome of metabolic surgery that improves PCOS. Meanwhile, some studies have suggested that alterations in the expression of inflammatory factors (5), gut hormones (6), and adipose hormones (7) contribute to the benefits of bariatric surgery in patients with PCOS. Sleeve gastrectomy (SG) is a popular bariatric surgical procedure; however, the suitability and potential mechanisms by which SG improves PCOS remain unknown.

To date, alterations in the gut microbiota and metabolites have been associated with the development of obesity and PCOS (8, 9). Numerous studies have indicated the changes in microbiota composition after SG, suggesting the relationship between shifts in the intestinal microbiota and metabolic improvement post-surgery (10). Modifications in the levels and types of several metabolites, particularly short-chain fatty acids (SCFAs), are considered to be associated with insulin resistance and dysfunctional steroid hormone management in PCOS (11). To this end, novel mechanisms have been proposed to explain the beneficial effects of SG on PCOS. However, the changes occurring in the intestinal flora and SCFA pool in patients with PCOS following SG need to be elucidated. We hypothesized that SG modifies the intestinal flora and SCFAs in patients with PCOS, and consequently, improves the outcomes of PCOS.

Here, we explored the potential effects of SG on the gut microbiota composition and alterations in SCFAs in a rat model of PCOS. Further, we investigated the association between the intestinal flora and efficacy of SG in PCOS.

## Methods

### Animals and Diet

All experiments were approved by the ethics committee of the Fujian Academy of Medical Sciences (#DL-2021-07). Twenty-one-day-old female Sprague-Dawley rats were purchased from Shanghai Laboratory Animals Center Co., Ltd. (Shanghai, China). All rats were housed individually and maintained under a 12 h light:dark cycle at 22 ± 2°C and 50%-60% humidity and were provided free access to food and water. Animal feed was obtained from Research Diets, Inc. (New Brunswick, NJ, USA). A standard rodent diet (D12450J, Research Diets; 3.85 kcal/g) is composed of 20% protein, 70% carbohydrate, and 10% fat. A high-fat diet (HFD, D12492, Research Diets; 5.24 kcal/g) is composed of 20% protein, 20% carbohydrate, and 60% fat. Dehydroandrosterone (HEA) was obtained from Cayman Chemical (Michigan, MI, USA).

### Experimental Design

Fifty-nine rats were randomly divided into three groups: (a) in the control group (CON, n = 15), rats were fed a normal rodent diet composed of 10% fat, and rats administered 0.2 mL tea oil were used as negative controls; (b) in the DHEA group (DHEA, n = 22), rats were fed a 10% fat diet and subcutaneously injected with DHEA (6 mg/100 g of body weight dissolved in 0.2 mL of tea oil for 21 consecutive days, administered daily (12, 13)); (c) in the DHEA+HFD group (DHF, n = 22), rats were fed a 60% HFD and treated according to the same schedule with DHEA subcutaneous injection. After 3 weeks of modeling, eight rats from each group were selected randomly for analyzing PCOS-related parameters. The remaining rats in the DHEA and DHF groups were randomly divided into the SG and sham surgery groups, whereas rats from CON underwent sham surgery. There were five groups for surgery: (1) CON+Sham group (CONSh, n = 7), (2) DHEA+Sham group (DHEASh, n = 7), (3) DHEA+SG group (DHEASg, n = 7), (4) DHF+Sham group (DHFSh, n = 7), (5) DHF+SG group (DHFSg, n = 7). DHEA was administered continuously *via* subcutaneous injection until the rats were euthanatized.

### Surgical Procedure

Surgery was performed on the 21^st^ day after DHEA injection. All rats were fasted for 12 h before surgery. Water was available ad libitum until 4 h before the intervention. The rats were anesthetized by administering 10% chloral hydrate (0.3 mL/100 g injected intraperitoneally).

SG was performed according to a previously published method (13) (Online Resource 1). To prevent potential interference with the gut flora, we avoided using antibiotics in this study.

In the first 24 h post-surgery, the rats were fasted without water and food to promote the healing of the newly stitched tresis vulnus. They were administered a liquid diet 24 h later, with gradual transition to a normal diet within 4-5 days post-surgery.

### Measurement of Hormones and Inflammatory Factors

The plasma levels of insulin, C-peptide, and the inflammatory factors tumor necrosis factor alpha (TNFα) and interleukin 6 (IL-6) were measured using the MILLIPLEX MAP RAT METABOLIC MAGNETIC BEAD PANEL KIT according to the manufacturer’s instructions (Merck Millipore, Billerica, MA, USA). The serum levels of testosterone, progesterone, and estradiol (E2) were measured using enzyme-linked immunosorbent assay kits (R&D Systems, Inc, Minneapolis, MN, USA). The serum levels of luteinizing hormone (LH) and follicle-stimulating hormone (FSH) were measured using a LUMINEX 200 (Luminex, Austin, TX, USA).

### Vaginal Smears and Ovarian Morphology Analysis

The vaginal smears of the rats were monitored daily to determine the stage of cyclicity from 6 weeks of age to the day before surgery and till 1 week post-surgery. The regular estrus cycle comprises four stages: proestrus, estrus, metestrus, and diestrus. After the rats were euthanized, the ovaries were harvested and fixed immediately with 10% formalin for 24 h. When the rats were anesthetized, the ovaries were rapidly removed from the animals. Adipose tissue was separated and bilateral ovaries were weighed. The left ovarian vesicle was fixed in 4% paraformaldehyde for later ovarian histomorphological examination. The right ovary was placed in a cryopreservation tube into liquid nitrogen for cryopreservation, and then stored at -80°C for later use. Hematoxylin and eosin (HE) staining was performed. The number of cystic follicles and corpora lutea (CL) was counted by two pathologists blinded to grouping.

### Body Weight Measurement and Glucose and Insulin Tolerance Tests

Each rat was weighed daily, starting at 28 days of age, until they were sacrificed. Glucose tolerance tests (GTTs) and insulin tolerance tests (ITTs) were conducted on the 18^th^ and 22^nd^ days post-surgery, respectively. The rats were fasted for 12 h. Blood from orbital veins was collected to measure the blood glucose level. For GTT, after the intraperitoneal injection of dextrose (2 g/kg body weight), the blood glucose levels were measured at 0, 15, 30, 45, and 60 min. For ITT, after the intraperitoneal injection of insulin (1 IU/kg body weight), the blood glucose levels were measured at 0, 30, 60, 90, and 120 min. GraphPad Prism 8.4 (GraphPad Software Inc., CA, USA) was used to calculate the total area under the glucose response curve (AUC). The serum levels of adiponectin and leptin were determined using enzyme linked immunosorbent assay (ELISA) kits (R&D Systems, Inc, Minneapolis, MI, USA).

### Sequencing and Analysis of Gut Microbiota

Fresh fecal samples were collected in 1.5-mL sterile Eppendorf tubes, snap-frozen in liquid nitrogen and stored at -80 °C for subsequent analyses. The sequences of the 16S rDNA hypervariable regions V3-V4 were amplified using the barcoded primers 341F 5’-CCTACGGGRSGCAGCAG-3’ and 806R 5’-GGACTACVVGGGTATCTAATC-3’ using the Illumina MiSeq platform (Online Resource 1). The composition of the gut microbial community was analyzed using Unweighted UniFrac of ANOSIM and principal coordinates analysis (PCoa). Next, to determine the differences in the community composition among the groups, we performed principal component analysis (PCA) at the genus level. To investigate the differences in the microbial composition between the SG and sham groups, we used the linear discriminant analysis effect size (LEfSe) method by coupling standard tests for statistical significance with additional analyses that evaluated the biological consistency and effect relevance.

### Fecal SCFA Measurement

Methanol was purchased from Sigma-Aldrich (St. Louis, MO, USA). Methyl chloroformate, acetonitrile, and isopropyl alcohol were obtained from Thermo Fisher Scientific (Fairlawn, NJ, USA). All standard chemicals required for the measurement of other microbial metabolites were obtained from Sigma-Aldrich, Steraloids Inc. (Newport, RI, USA), and TRC Chemical (Toronto, ON, Canada). Ultrapure water was obtained using a Mill-Q Reference system equipped with an LC-MS Pak filter (Millipore, Billerica, MA, USA) (Online Resource 1). In order to analyze changes in the composition of metabolites related to the gut microbiota composition, we conducted ultra-performance liquid chromatography-tandem mass spectrometry analysis to measure the metabolite content.

### Statistical Analysis

Statistical analysis was performed using GraphPad Prism 8.4, SPSS 24.0 (IBM Corp., NY, USA), and R (version 3.6.3). Experimental data are expressed in terms of mean ± standard deviation from at least three independent experiments. Data were analyzed using one-way analysis of variance to compare the mean values of variables from different groups. Bonferroni’s test or Tukey’s post-hoc test was performed for the pairwise comparison of mean values among the groups. Spearman’s correlation analysis was performed to identify the correlations among microbiotas. Pearson’s correlation analysis was performed to determine the correlation among clinical phenotypes, gut microbiota, and fecal metabolites. P < 0.05 was considered to represent statistical significance.

## Results

### SG Decreased the Abundance of Gut Microbiota in the DHEASg Group but Not in the DHFSg Group

**Figure 1:**
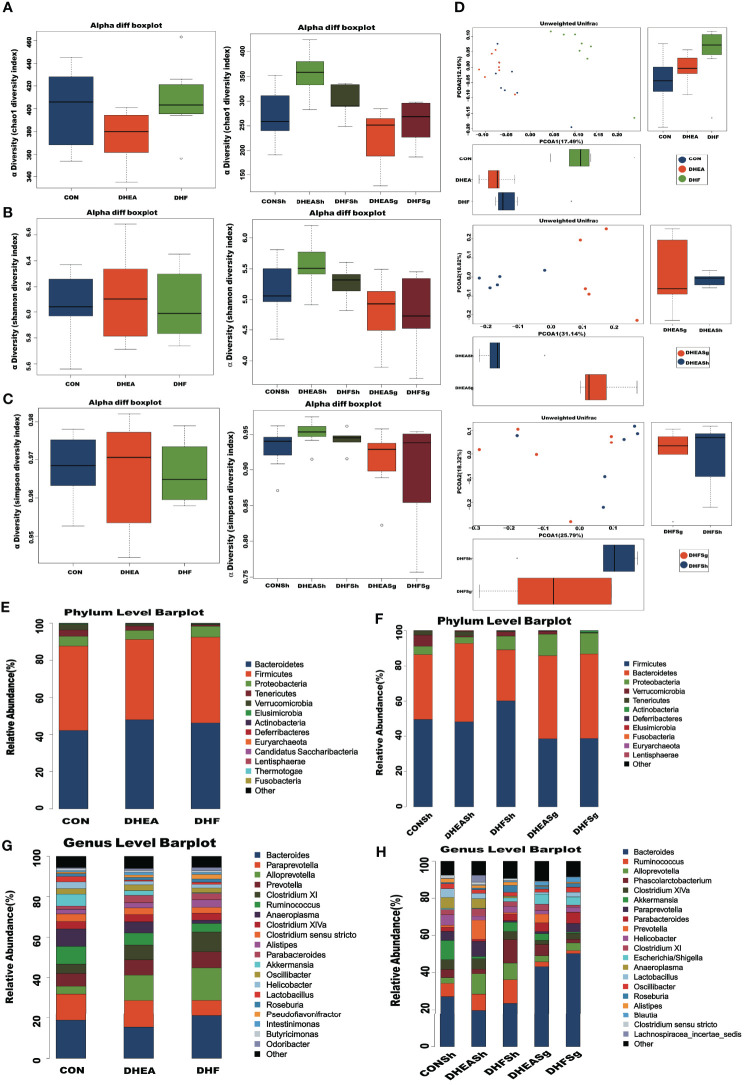
Effects of SG on shifts in gut microbiota. **(A)** Chao1 diversity index. **(B)** Shannon diversity index. **(C)** Simpson diversity index. **(D)** β diversity analysis using Unweighted UniFrac ANOSIM analysis and principal coordinates analysis (PCA) of bacterial community composition using the unweighted UniFrac metric at the genus level. **(E-H)** Bar plot of the microbial community structure at the phylum level before **(E)** and after **(F)** surgery, and at the genus levels before **(G)** and after **(H)** surgery.

A value of 0.99 was obtained for the Good’s coverage index, which indicated that the data was sufficient for analyzing the intestinal microbial community using the study samples (**Supplementary Figure 1A**). The Chao1 index indicated the richness of the gut microbial community. There were no significant differences between the Chao1 indices of CON and PCOS rats pre-surgery or between those of DHFSg and DHFSh rats post-surgery (**Figure 1A**). However, the Chao1 index was lower in the DHEASg group than in the DHEASh group (**Figure 1A**). The Shannon and Simpson indices indicated the diversity and evenness of intestinal flora. There were no significant differences between the parameters obtained for the CON and PCOS groups (**Figures 1B, C**) or those obtained for the corresponding SG and sham surgery groups (**Figures 1B, C**). The results showed that SG did not affect the α diversity of the gut microbiota. Interestingly, SG decreased the abundance of the gut microbiota in the DHEASg group but not in the DHFSg group (**Figures 1A–C**).

### SG Drastically Altered the Abnormal Gut Microbial Composition in the Rat Model of PCOS

The PCoA results indicated a significant difference among species present in fecal samples from the CON, DHEA, and DHF groups (**Figure 1D** and **Supplementary Figure 1B**). The intestinal microbiota in the CON and PCOS groups were distinct with respect to the organismal structure. Notably, the PCoA plots revealed a distinct separation between the SG and sham surgery groups, suggesting that SG altered the gut microbiota composition in rats with PCOS (**Figure 1D**). Besides, ANOSIM analysis yielded similar results (**Supplementary Figure 1B**).

### SG Restored Gut Dysbiosis in Rats With PCOS by Altering the Abundances of Specific Taxa

At the phylum level (**Figures 1E, F**), *Bacteroidetes*, *Firmicutes*, and *Proteobacteria* were the major microbial taxa detected in the groups. The abundance of *Firmicutes* decreased in both DHEASg and DHFSg rats compared to that in the corresponding sham groups; this change could be attributed to SG. Furthermore, the abundance of *Bacteroidetes* increased in DHEASg and DHFSg rats. At the genus level (**Figures 1G, H**), we analyzed the top twenty bacteria. The abundances of *Bacteroides* and *Blautia* increased more in the SG groups than in the sham groups, whereas the relative abundances of *Ruminococcus*, *Alloprevotella*, *Clostridium*, and *Alistipes* were low.

**Figure 2:**
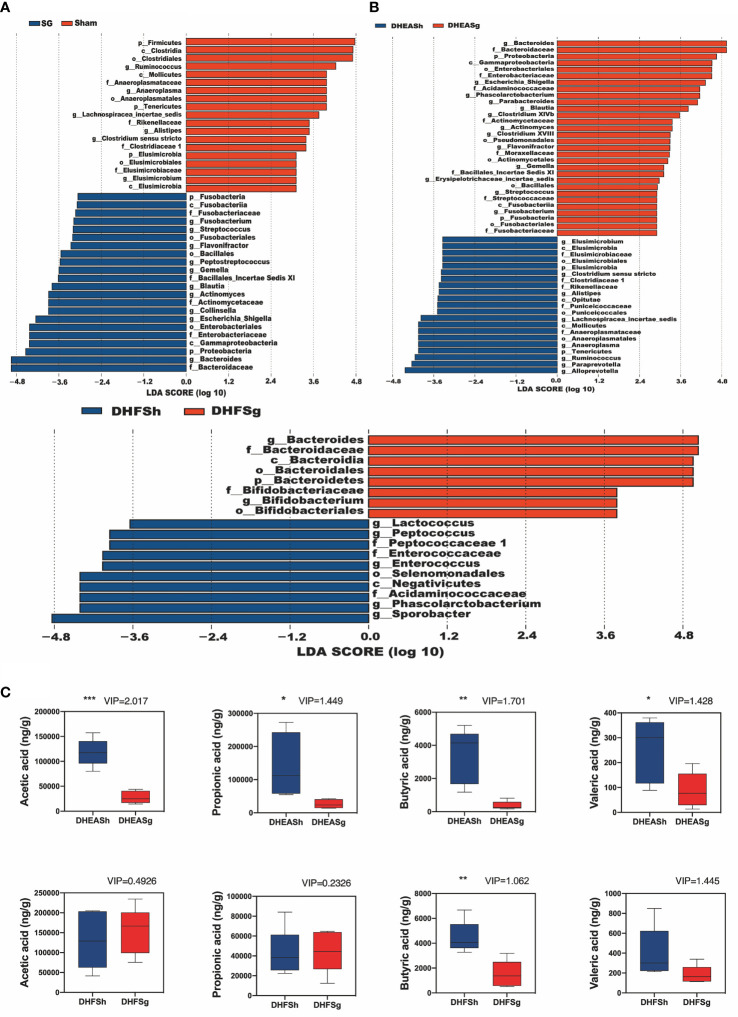
Effects of SG on shifts in gut microbiota and short chain acid contents. **(A)** LDA effect size (LEfSe) analysis between the SG and sham groups and between the DHEASg and DHEASh groups. **(B)** LEfSe analysis between the DHFSg and DHFSh groups. **(C)** Boxplots show changes in the levels of acetic acid, propionic acid, butyric acid, and valeric acid. *p < 0.05. **p < 0.01. ***p < 0.001.

Furthermore, the abundances of Bacteroides, Escherichia_Shigella, Collinsella, Actinomyces, Blautia, Gemella, Peptostreptococcus, Flavonifractor, Streptococcus, and Fusobacterium increased in the PCOS-SG group. In contrast, the abundances of Ruminococcus, Alistipes, Anaeroplasma, Lachnospiracea_incertae_sedis, and Elusimicrobium increased significantly in the DHEASh and DHFSh groups (**Figure 2A** and **Supplementary Figure 1C**). Compared to the rats from the DHEASh group, those from the DHEASg group had a greater abundance of Bacteroides, Blautia, and Parabacteroides at the genus level (**Figure 2A** and **Supplementary Figure 1D**). However, the abundances of Alistipes, Ruminococcus, Paraprevotella, and Alloprevotella were notably lower in the DHEASg group (**Figure 2A** and **Supplementary Figure 1D**). Additionally, the abundances of Bacteroides and Bifidobacterium increased and those of Lactococcus, Peptococcus, and Enterococcus decreased considerably in the DHFSg group than in the DHFSh group (**Figure 2B** and **Supplementary Figure 1D**). Therefore, the abundances of microbial species in the SG and sham groups differed considerably.

### SG Markedly Increased the Levels of Fecal SCFAs, Particularly Butyric and Valeric Acids, in Rats With PCOS

PCA is primarily applied to analyze the chief source of alterations among samples. An observable distance between the PCOS (DHEA and DHF) and CON groups suggested that PCOS disturbed the fecal metabolite composition in rats (**Supplementary Figure 2A**). Based on the PCA results, the SG group was found to be metabolically distant from the corresponding groups, indicating that SG modulated the metabolic disturbance in rats with PCOS (**Supplementary Figure 2A**). Next, we conducted orthogonal partial least squares discriminant analysis (OPLS-DA) to maximize differences and filter core metabolites reliable for categories among groups. The generated models rendered a reliable predictive ability (DHEASg-DHEASh:R2Y = (0.0,0.9533), Q2 = (0.0, −0.1709) (**Supplementary Figure 2B**), and DHFSg-DHFSh:R2Y = (0.0,0.9391), Q2 = (0.0, −0.058) (**Supplementary Figure 2B**)). Based on the values of variable influence on projection > 1.0, both the DHEASg and DHFSg groups were found to exhibit a significant shift in the levels of butyric and valeric acids compared to those in the corresponding sham surgery groups (**Figure 2C**). However, the levels of acetic acid and propionic acid were reduced significantly only in the DHEASg group. These findings indicated that SG-induced alterations in gut microbiota may markedly increase the fecal levels of SCFAs, particularly butyric and valeric acids, in rats with PCOS (**Figure 2** and **Supplementary Figure 2**).

### SG Considerably Ameliorated Ovarian Dysfunction in PCOS

**Figure 3:**
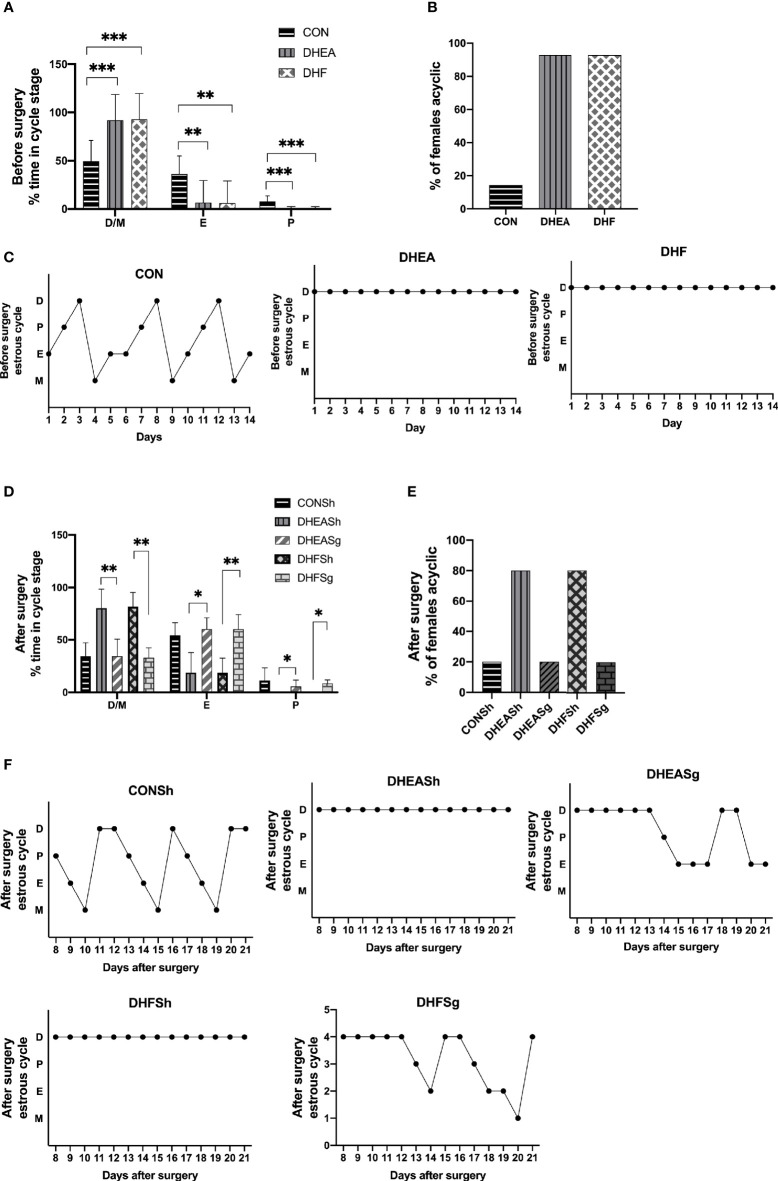
Effects of sleeve gastrectomy on the estrus cycle. **(A)** Relative time span of each stage of the cycle pre-surgery. **(B)** The proportion of acyclic females (constant diestrus or constant estrus) pre-surgery. **(C)** Representative estrus cycles of different groups pre-surgery: 1, metestrus stage; 2, estrus stage; 3, proestrus stage; 4, diestrus stage. C-a: CON; C-b: DHEA; C-c: DHF. **(D)** Relative time span of each stage of the cycle post-surgery. **(E)** The proportion of acyclic females (constant diestrus or estrus) post-surgery. **(F)** Representative estrus cycles of different groups post-surgery. F-a: CONSh; F-b: DHEASh; F-c: DHEASg; F-d: DHFSh; F-e: DHFSg. *p < 0.05. **p < 0.01. ***p < 0.001.

**Figure 4:**
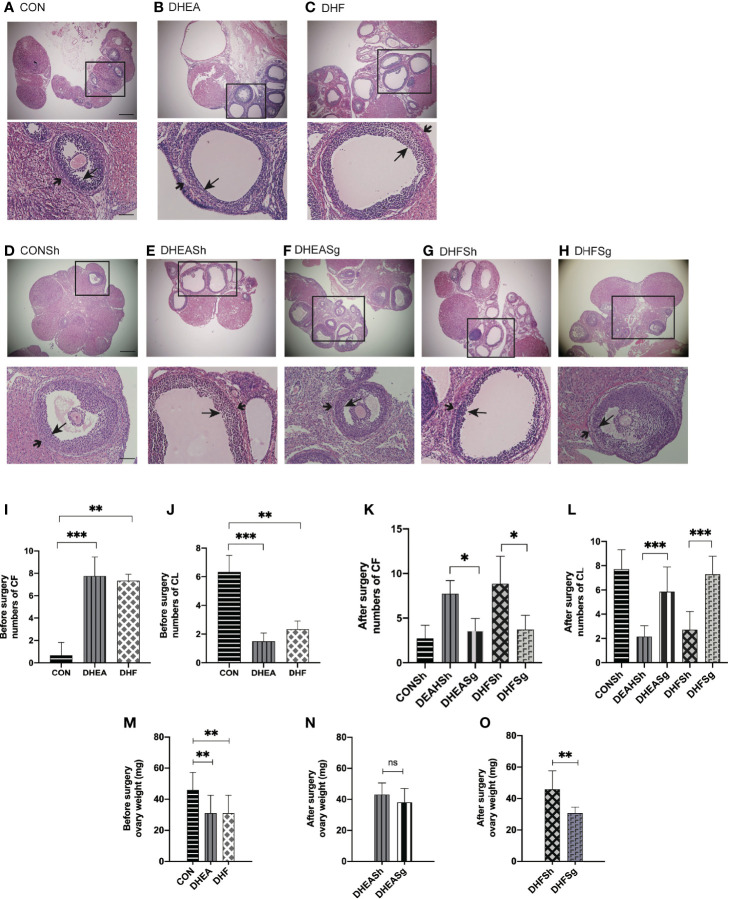
Effects of sleeve gastrectomy (SG) on ovarian morphology, as observed in hematoxylin and eosin-stained tissue sections. **(A, B)** Representative images of ovarian tissues from CON **(A)**, DHEA **(B)**, and DHF **(C)** rats pre-surgery. **(D–H)** Representative images of ovarian tissues from CONSh **(D)**, DHEASh **(E)**, DHEASg **(F)**, DHFSh **(G)**, and DHFSg **(H)** rats post-surgery. From **(A–H)**: Scale bar, 100 μm; original magnification for a, b, and c: ×4, ×10, and ×20, respectively. The short arrows indicate the theca, and the long arrows indicate the granulosa layer. **(I, J)** The number of CF **(I)** and CL **(J)** before SG. **(K, L)** The number of CF **(K)** and CL **(L)** after SG. CF, cystic follicle; CL, corpus luteum. **(M)** Ovary weight before surgery. **(N)** Ovary weight after surgery in the DHEASh and DHEASg groups. **(O)** Ovary weight after surgery in the DHFSh and DHFSg groups. *p < 0.05. **p < 0.01. ***p < 0.001.

Analysis of the estrus cycle revealed that both PCOS models experienced a significantly longer diestrus and shorter proestrus and estrus than CON rats (**Figures 3A–C**). Intriguingly, SG intervention alleviated the estrus cycle disorder in rats with PCOS (**Figures 3D, E**). In the DHEA and DHF rats, which were acyclic prior to surgery, the regular estrus cycle was restored from 12 days after SG (**Figure 3F**). However, the DHEASh and DHFSh rats continued to exhibit an irregular cycle throughout the intervention period (**Figure 3F**). DHEA and DHF rats had polycystic ovaries, whereas CON and PCOS rats did not. Multiple ovarian follicles from DHEA and DHF rats showed the presence of a tangled granulosa cell compartment with abnormal granulosa layer thickness, a typical characteristic of atretic antral follicles (**Figures 4A–C**). Notably, the ovaries of DHEA and DHF rats lacked CL, whereas the ovaries of CON rats had multiple CL, indicative of ovulation (**Figures 4I–L**). HE staining revealed that the granulosa layers of ovarian tissue from both PCOS-Sham groups showed a reduced number of cystic degenerating follicles. In contrast, SG increased the formation of granulosa layers and CL, indicating that SG improved the pathological changes observed in PCOS (**Figures 4D–H, K, L**). After modeling, the left ovaries of rats in each group were weighed, as shown in **Figures 4M–O**. After DHEA injection, the ovarian weight was lower than that of the control group, regardless of whether the diet was high-fat or not. Compared with the DHFSh group, the ovarian weight of PCOS rats in DHFSg group was lower. While there were no differences in ovarian weights between the DHEASh and DHEAsg groups.

### SG Considerably Improved the Levels of Sex Steroid Hormones and Inflammatory Factors in Rats With PCOS

**Figure 5:**
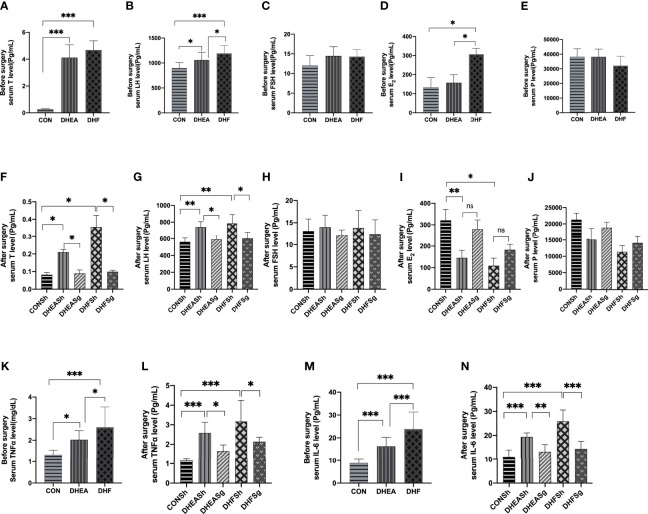
**|** Effects of sleeve gastrectomy on the serum levels of steroid sex hormones and inflammatory factors. **(A)** total testosterone, **(B)** LH, **(C)** FSH, **(D)** E2, and **(E)** progesterone levels pre-surgery. **(F)** total testosterone, **(G)** LH, **(H)** FSH, **(I)** E2, and **(J)** progesterone levels post-surgery. **(K)** serum TNFα levels pre-surgery, **(L)** serum TNFα levels post-surgery, **(M)** serum IL-6 levels pre-surgery, **(N)** serum IL-6 levels post-surgery. LH: luteinizing hormone, FSH: follicle-stimulating hormone, E2: estradiol, TNFα: tumor necrosis factor alpha, IL-6: interleukin 6. *p < 0.05. **p < 0.01. ***p < 0.001.

Hyperandrogenism is the most prominent feature of PCOS. The serum testosterone levels increased considerably in DHEA and DHF rats than in CON rats (**Figure 5A**), indicating hyperandrogenism in the former. In addition, DHEA injection, with or without HFD, increased the serum levels of LH but not of FSH and progesterone (**Figures 5B–D**). Notably, both testosterone and LH levels were reduced, and almost restored to the normal levels, in DHEASg and DHFSg rats (**Figures 5F, G**). In addition, the circulating levels of TNFα and IL-6 were significantly elevated in both DHEA and DHF rats but were normalized upon SG intervention (**Figures 5K–N**). The above results confirmed that SG treatment considerably improved the levels of sex steroid hormones and inflammatory factors in PCOS (**Figure 5**).

### SG Reduced Body Weight and Insulin Resistance in PCOS

**Figure 6:**
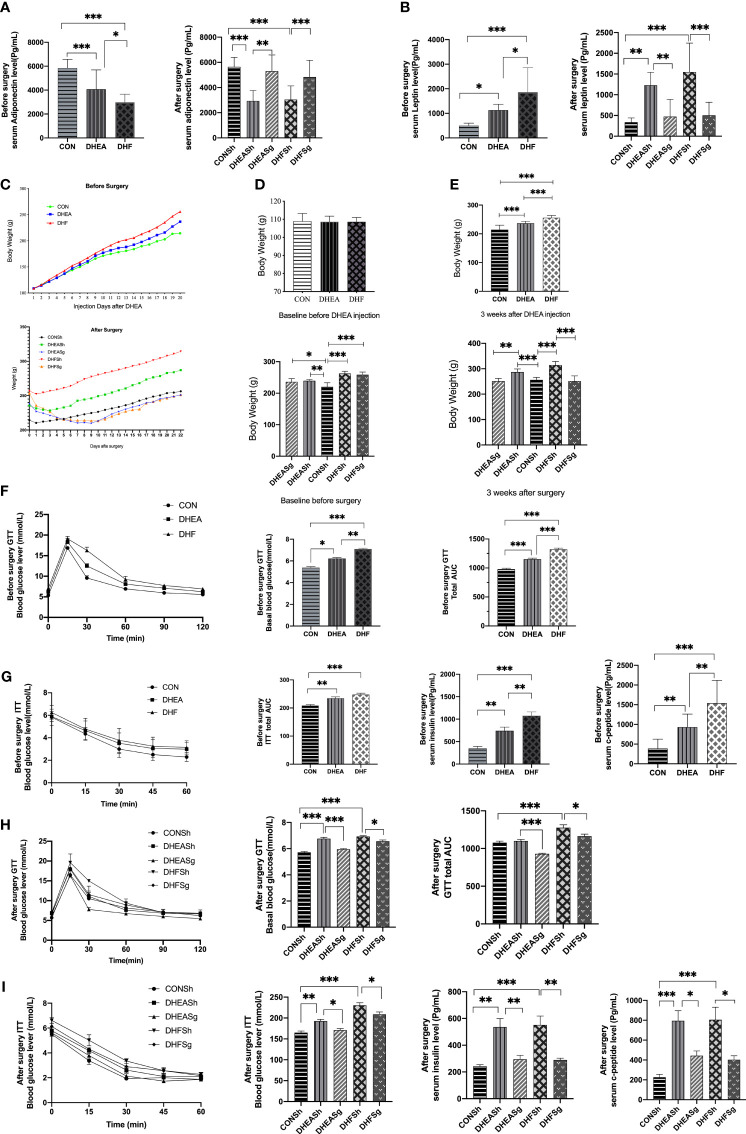
Effects of sleeve gastrectomy on the metabolic hallmarks of polycystic ovary syndrome. Changes in adiponectin **(A)** and leptin **(B)** before and after surgery. **(C)** Changes in body weight before and after surgery. **(D)** Changes in body weight before DHEA injection and before surgery. **(E)** Changes in body weight 3 weeks after DHEA injection and 3 weeks after surgery. **(F)** Results of intraperitoneal glucose tolerance test and **(G)** intraperitoneal insulin tolerance test before surgery. **(H)** Results of intraperitoneal glucose tolerance test and **(I)** intraperitoneal insulin tolerance test after surgery. Serum c-peptide levels before **(G)** and after **(I)** surgery. *p < 0.05. **p < 0.01. ***p < 0.00`1.

We detected the expression level of adiponectin, the results showed adiponectin was remarkably decreased in PCOS models compared with control rats (**Figure 6A**). The serum level of adiponectin was restored in DHEASg group and in DHFSg group compared to their corresponding sham operation group (**Figure 6A**). Inversely, leptin was significantly elevated both in DHEA and DHF rats, but were normalized by SG (**Figure 6B**). Moreover, DHF-treated rats showed a remarkably higher levels of leptin than DHEA-treated rats (**Figure 6B**). The DHEA and DHF rats gained more weight than CON rats during the 3-week treatment period (**Figures 6C–E** and **Supplementary Figure 3A**). Post-surgery, both DHEASg and DHFSg rats lost significantly more weight than DHEASh and DHFSh rats, starting 1 week post-surgery, and maintained a lower body weight till the end of the experiment (**Figures 6C–E** and **Supplementary Figure 3B**). Glucose homeostasis is typically impaired in patients with obesity and metabolic syndrome, including patients with PCOS. We found that the basal glucose levels were elevated in both DHEA and DHF rats (**Figure 6F**). Moreover, both DHEA and DHF rats exhibited impaired insulin sensitivity (**Figure 6G**). Post-surgery, the AUC for the SG excision group was significantly lower than that for the sham surgery and CON groups. As expected, both insulin sensitivity and glucose tolerance were significantly improved in DHFSg and DHEASg rats compared to that in the sham controls (**Figures 6H, I**). These results indicated the significant benefits of SG intervention on insulin resistance (**Figure 6**).

### Gut Bacteria, Sex Steroid Hormones, Inflammation, and Fecal SCFAs Exhibit Close Correlation Following SG in Rats With PCOS

**Figure 7:**
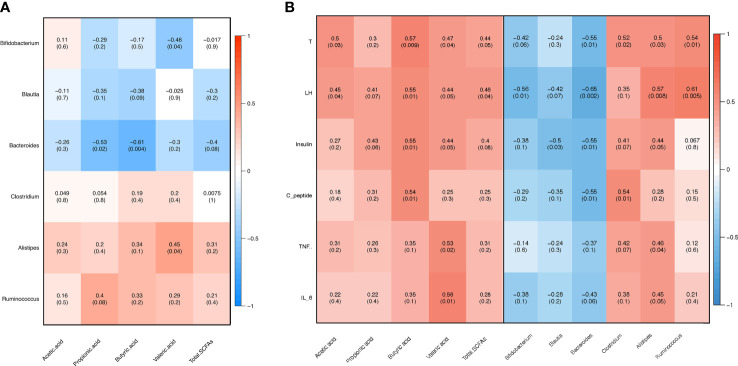
Pearson’s correlation analysis. **(A)** Correlation between fecal gut microbiota and short-chain fatty acids (SCFAs). **(B)** Correlation between clinical variables and gut microbiota as well as fecal SCFAs. T: testosterone, LH: luteinizing hormone, FSH: follicle-stimulating hormone, TNFα: tumor necrosis factor alpha, IL-6: interleukin 6.

In order to assess the association among gut microbiota, inflammation, sex steroid hormones, and fecal SCFAs, we performed a correlation analysis. The abundances of *Bacteroides*, *Blautia*, and *Bifidobacterium* were negatively correlated with the fecal SCFA content, whereas those of *Alistipes* and *Ruminococcus* were positively correlated with the fecal SCFA content (**Figure 7A**). The abundance of *Bacteroides* exhibited a significant negative correlation with the butyric acid, propionic acid, and total SCFA content. Conversely, the levels of LH and testosterone were positively associated with the levels of SCFAs, including butyric acid, propionic acid, acetic acid, valeric acid, and total SCFAs (**Figure 7B**). The levels of butyric acid were positively associated with those of insulin and C-peptide. Moreover, the abundances of *Bacteroides* and *Blautia* were negatively correlated with the LH, testosterone, insulin, and c-peptide levels (**Figure 7B**). In contrast, the abundances of *Alistipes* and *Ruminococcus* were positively correlated with the LH and testosterone levels. The abundance of *Bacteroides* was negatively associated with the IL-6 levels, the relative abundance of *Clostridium* was positively correlated with the TNFα and IL-6 levels, and the abundance of *Alistipes* was only positively associated with the TNFα level (**Figure 7**).

## Discussion

To our knowledge, the findings of this study indicated for the first time that SG-induced changes in the gut microbiota and SCFAs may contribute to the beneficial effects of SG on PCOS.

It is well known that one of the major challenges in investigating PCOS in a rat model is the heterogeneity of phenotypes that PCOS patients present. Different phenotypes of PCOS show different characteristics of this disorder (14). In this study, the DHEA-treated rodents exhibited some symptoms of PCOS, including hyperandrogenism, disrupted menstrual cycle, and polycystic ovaries, which was in accordance with previous studies (14, 15). In addition, the HFD further exaggerated metabolic dysfunction, which typically resulted in obesity and insulin resistance in rats of the DHF group. Thus, two types of PCOS rat models were observed: one was dominated by hyperandrogenism, the other was characterized by metabolic dysfunction. It is worthy of note that there was a marked decrease in the ovarian weight when large doses of DHEA was administered. As early as in 1962, Roy et al. (16) found that the ovarian weight of rats was reduced after the administration of large dose of DHEA. It is not known whether the uterotrophic action of these steroids is due to a direct effect or to their conversion into testosterone or oestrogen.

Alterations in the intestinal microbiota and microbial metabolites and their potential interactions following SG in patients with PCOS is a topic of general interest. In order to take into account that the high-fat diet itself has a certain effect on the intestinal flora and short-chain fatty acids, the feed for all groups of rats after the operation is the same as that before the operation. However, data on the effects of SG on the gut microbiome and SCFAs associated with metabolic and physiological changes in patients with PCOS are lacking. Here, we investigated the influence of SG on the gut microbiota composition and SCFA content in DHEA-induced PCOS rat models. In agreement with the findings from previous studies, we observed a reduction in the abundance of *Firmicutes* and a shift in favor of *Bacteroidetes* in response to SG in the DHEA-induced rat model (14, 15). Reportedly, *Firmicutes* can facilitate fat storage in the host body (17). Therefore, the decreased abundance of *Firmicutes* may help reduce fat storage after SG. Meanwhile, the elevated abundance of *Bacteroidetes* was considered associated with weight loss in humans and animal models (10, 18, 19).

Furthermore, at the genus level, the abundances of *Bacteroides* and *Blautia* increased considerably in the PCOS-SG groups. Rats in the PCOS-SG groups also exhibited improved glucose intolerance. The abundance of *Bacteroides* was negatively correlated with the insulin and C-peptide levels. Liu et al. showed that *Bacteroides* can protect mice against HFD-induced obesity (19). Therefore, we speculated that *Bacteroides* are associated with metabolic amelioration in PCOS after SG. Our correlation analysis of different bacteria at the genus level also showed that *Blautia* and *Bacteroides* exhibit a synergistic relationship*. Blautia* is known to produce butyric acid in the gut, which may potentially reduce the risk of metabolic syndrome (20). The abundance of *Bifidobacterium* increased predominantly in the DHFSg group but not in the DHEASg group. As a beneficial bacterium, *Bifidobacterium* has been shown to improve insulin sensitivity and reduce body fat accumulation in several human randomized control trials (21–23). We also observed a lower abundance of *Ruminococcus, Clostridium*, and *Alistipes* in PCOS-SG rats. *Ruminococcus* and *Clostridium* were shown to be enriched patients with PCOS (24) and rodent models of PCOS (25). Hyperandrogenism is another characteristic of PCOS. *Ruminococcus* and *Clostridium* were positively associated with the serum levels of testosterone, which is consistent with the results from previous studies (26, 27). Hence, the marked decrease in the abundances of *Ruminococcus* and *Clostridium* following SG may be correlated with the amelioration of reproductive disorder in rats with PCOS.

Numerous studies have indicated that chronic low-grade inflammation contributes to PCOS development (21, 28). The correlation analysis between gut microbiota and inflammatory factors indicated that the elevated relative abundances of *Clostridium* and *Alistipes* were related to gut inflammation (29). A higher abundance of *Alistipes* was reported in patients with irritable bowel syndrome (29). In this study, the low levels of TNFα and IL-6 suggested the alleviation of inflammation following SG in rats with PCOS. Moreover, the relative abundances of *Clostridium* and *Alistipes* were positively correlated with the TNFα levels. Conversely, the abundance of *Bacteroides* was negatively associated with the IL-6 levels. Collectively, our data confirmed the alterations in the gut microbiota of DHEA-induced rat models in response SG. The increasing abundances of *Bacteroides* and *Blautia* and the decreasing abundances of *Ruminococcus*, *Clostridium*, and *Alistipes* in response to SG may play a central role in the remodeling of the intestinal microbiota in PCOS. Our findings indicated that the alterative gut microbiome community was closely associated with the improvement of hyperandrogenism, lowering of inflammation, and alleviation of insulin resistance.

Besides modeling the gut microbiome, SG also altered the production of metabolites such as SCFAs. SCFAs are primarily produced during bacterial fermentation in the gut. Acetic, propionic, butyric, and valeric acids are the predominant SCFAs detected in the gut of rodents and humans (30). Common bacteria that produce SCFAs include, but are not limited to, *Bacteroides* and *Bifidobacterium* (31). SCFAs were shown to improve energy metabolism and exert strong anti-inflammatory effects (32, 33). In this study, we measured the fecal concentrations of SCFAs as substitutes for evaluating SCFA production in the gut. The fecal concentrations of total or individual SCFAs depended on the production, mucosal absorption, or the rate of transit. Of note, the fecal SCFA content indicates the balance between the production and absorption of SCFAs (34).

We observed an increase in the abundance of *Bacteroidetes* following SG in both obese and lean phenotypes of rats with PCOS, whereas the abundance of *Bifidobacterium* increased significantly only in obese PCOS-SG rats. However, the fecal SCFA concentration, which was higher in rats with PCOS, decreased significantly after SG. Furthermore, the fecal SCFA content was positively correlated with the serum levels of testosterone and LH. The inverse association indicates the potential increase in the post-operative colonic absorption of SCFAs in rats with PCOS. The availability of SCFAs accelerates intestinal transit and systemic absorption of nutrients while optimizing energy utilization. In contrast, the greater demand for SCFAs increased the abundance of SCFA-producing bacteria, such as *Bacteroidetes* and *Bifidobacterium.*

In this regard, we speculated that the host sustained itself in a negative energy expenditure mode post-surgery. There may exist an adaptive response to the insufficient energy availability in the colon that involves SCFA production. The intestinal transit of gut SCFAs, particularly butyric acid, was accelerated to improve nutrient absorption. The higher SCFA utilization rate following SG treatment improved the intestinal mucosal barrier integrity and suppressed enteral and parenteral inflammation. Therefore, even though the fecal SCFA levels were positively associated with the TNFα and IL-6 levels, the changes in SCFA levels were possibly related to alterations in the abundances of certain microbial taxa following SG intervention, which may have helped reduce inflammation in the rats with PCOS.

However, the changes in the abundance and composition of gut microbiota were partially different between DHEASg and DHFSg groups. We consider that the different changes in the abundance and composition of gut microbiota between the two models is partly due to the differences in the establishment of the two PCOS models, DHEA-model is induced by DHEA injection alone, while the DHF model is induced by DHEA injection combined with a high-fat diet. Taking into account that the high-fat diet itself has a certain effect on the intestinal flora, such as overall gut microbial composition and the composition of the microbial community in gut (27), which may leads to the differences of gut microbiota between two PCOS groups. However, the same changes in gut microbiota of the two different models suggests that PCOS may have the common mechanism, which maybe a new therapeutic target.

The strength of this study is that is used two different models of PCOS and that it comprehensively analyzed all bacterial species. Still, the potential limitations to this study include the lack of adequate blood samples for the measurement of SCFA levels and the absence of germ-free animals for demonstrating the causal relationships between the gut microbiota and PCOS. The time-dependent alterations of the gut microbiota and SCFAs following SG should be investigated further. In addition, the study was designed to compare each model to its own control, not to compare the models between them.

This is the first study to demonstrate that SG modified the gut microbiome and SCFA content in a DHEA-induced rat model of PCOS. The altered abundances of bacterial taxa in response to SG may play a central role in the remodeling of the intestinal microbiome in rats with PCOS. The concentration of fecal SCFAs, especially butyric acid, which increased in the rats with PCOS, reduced distinctly after SG. Furthermore, the amelioration of PCOS-related reproductive and metabolic disorders in response to SG was associated with the regulation of specific microbial taxa and SCFAs. Our findings provide a new perspective on the changes in microbial mechanisms in PCOS after SG.

## Data Availability Statement

The original contributions presented in the study are publicly available. This data can be found here: https://doi.org/10.6084/m9.figshare.16832659.v1.

## Ethics Statement

All experimental procedures were approved by the ethics committee of the Fujian Academy of Medical Sciences (#DL-2021-07), according to the national legislation for animal care.

## Author Contributions

All authors contributed to the study conception and design. Material preparation was performed by WL and LW. Data collection and analysis were performed by WL and JW. Conception and design of the study were performed by WL, JW and GX. The first draft of the manuscript was written by WL, and all authors commented on previous versions of the manuscript. All authors contributed to the article and approved the submitted version.

## Funding

This study was supported by the Natural Science Foundation of Fujian Province (Grant number 2019J01175), the Young and Middle-aged Talents Training Project of Fujian Provincial Health Commission (Grant number 2018-ZQN-1) and Natural Science Foundation of China (Grant number 81770848, 82070878). The funders had no role in study design, data collection and analysis, decision to publish, or preparation of the manuscript.

## Conflict of Interest

The authors declare that the research was conducted in the absence of any commercial or financial relationships that could be construed as a potential conflict of interest.

## Publisher’s Note

All claims expressed in this article are solely those of the authors and do not necessarily represent those of their affiliated organizations, or those of the publisher, the editors and the reviewers. Any product that may be evaluated in this article, or claim that may be made by its manufacturer, is not guaranteed or endorsed by the publisher.

## Supplementary Material

Supplementary Figure 1 Effects of SG on shifts in gut microbiota. **(A)** α diversity analysis using Goods_coverage diversity index. **(B)** β diversity analysis using Unweighted UniFrac ANOSIM analysis and principal coordinates analysis (PCA) of bacterial community composition using the unweighted UniFrac metric at the genus level. **(C)** Spearman’s correlation analysis of differential bacterial groups at the genus level. **(D)** PCA between DHEASg and DHEASh groups and between DHFSg and DHFSh groups.

Supplementary Figure 2 Effects of sleeve gastrectomy (SG) on the alterations in gut metabolite composition. **(A)** Principal coordinates analysis of fecal metabolites. **(A)** CON, DHEA, and DHF; **(B)** DHEAsg and DHEASh; **(C)** DHFSg and DHFSh. **(B)** OPLS-DA analysis. **(A)** permutations in DHEASg and DHEAsh; **(B)** OPLS-DA plot between DHEASg and DHEAsh groups; **(C)** permutations in DHFSg and DHFSh; d OPLS-DA plot between DHFSg and DHFSh groups.

Supplementary Figure 3 Changes in body weight before **(A)** and after **(B)** surgery. *p < 0.05. **p < 0.01. ***p < 0.001.

Supplementary Information Supplementary methods (.doc).
