# Supplementary figures and images for: Effects of Sleeve Gastrectomy on Fecal Gut Microbiota and Short-Chain Fatty Acid Content in a Rat Model of Polycystic Ovary Syndrome
Source: Front Endocrinol (Lausanne). 2021 Nov 11;12:747888. doi: 10.3389/fendo.2021.747888 (PMC8631770; doi:10.3389/fendo.2021.747888)

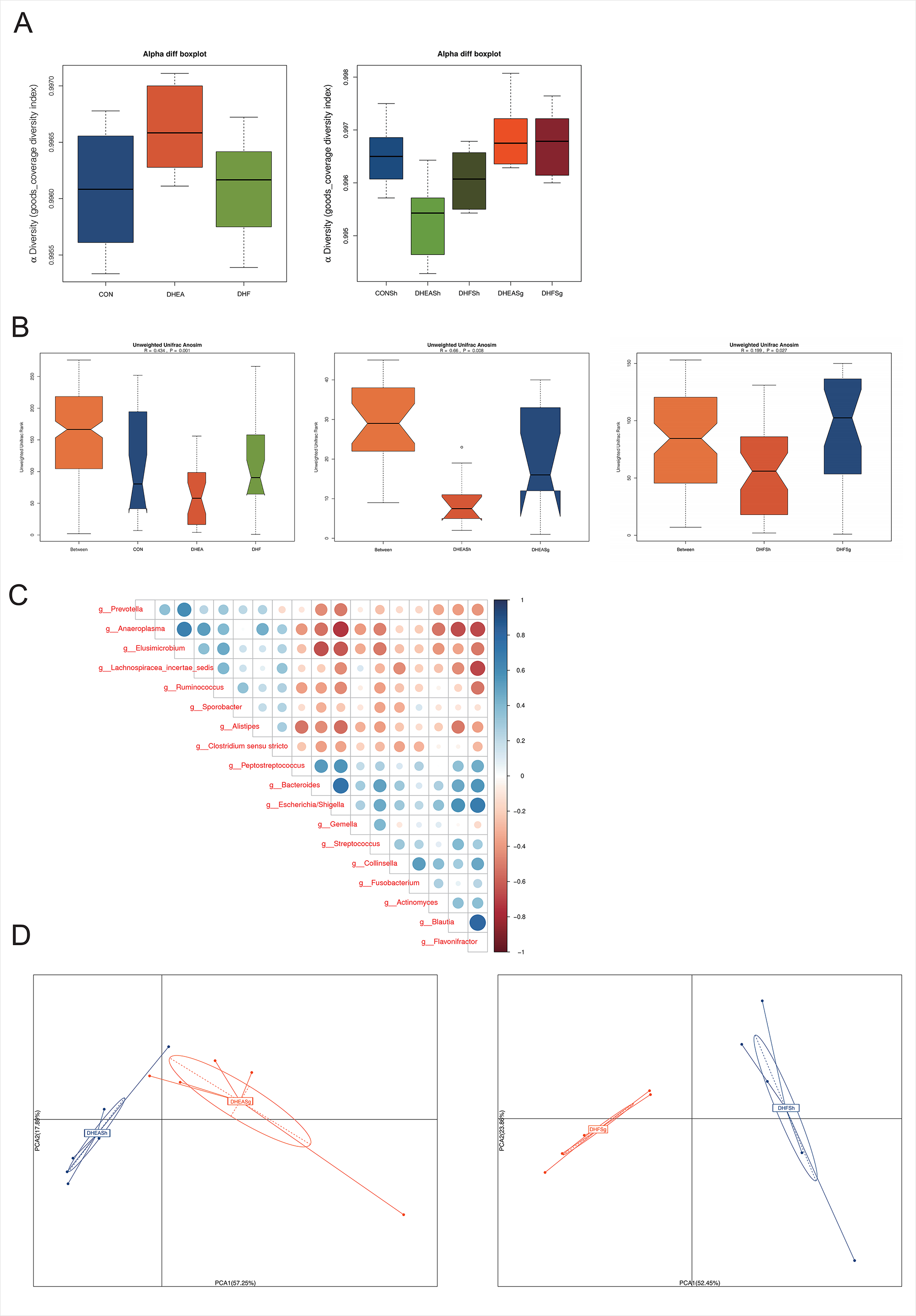

Supplement: Supplementary Figure 1 — Effects of SG on shifts in gut microbiota. (A) α diversity analysis using Goods_coverage diversity index. (B) β diversity analysis using Unweighted UniFrac ANOSIM analysis and principal coordinates analysis (PCA) of bacterial community composition using the unweighted UniFrac metric at the genus level. (C) Spearman’s correlation analysis of differential bacterial groups at the genus level. (D) PCA between DHEASg and DHEASh groups and between DHFSg and DHFSh groups. [file Image_1.tif]

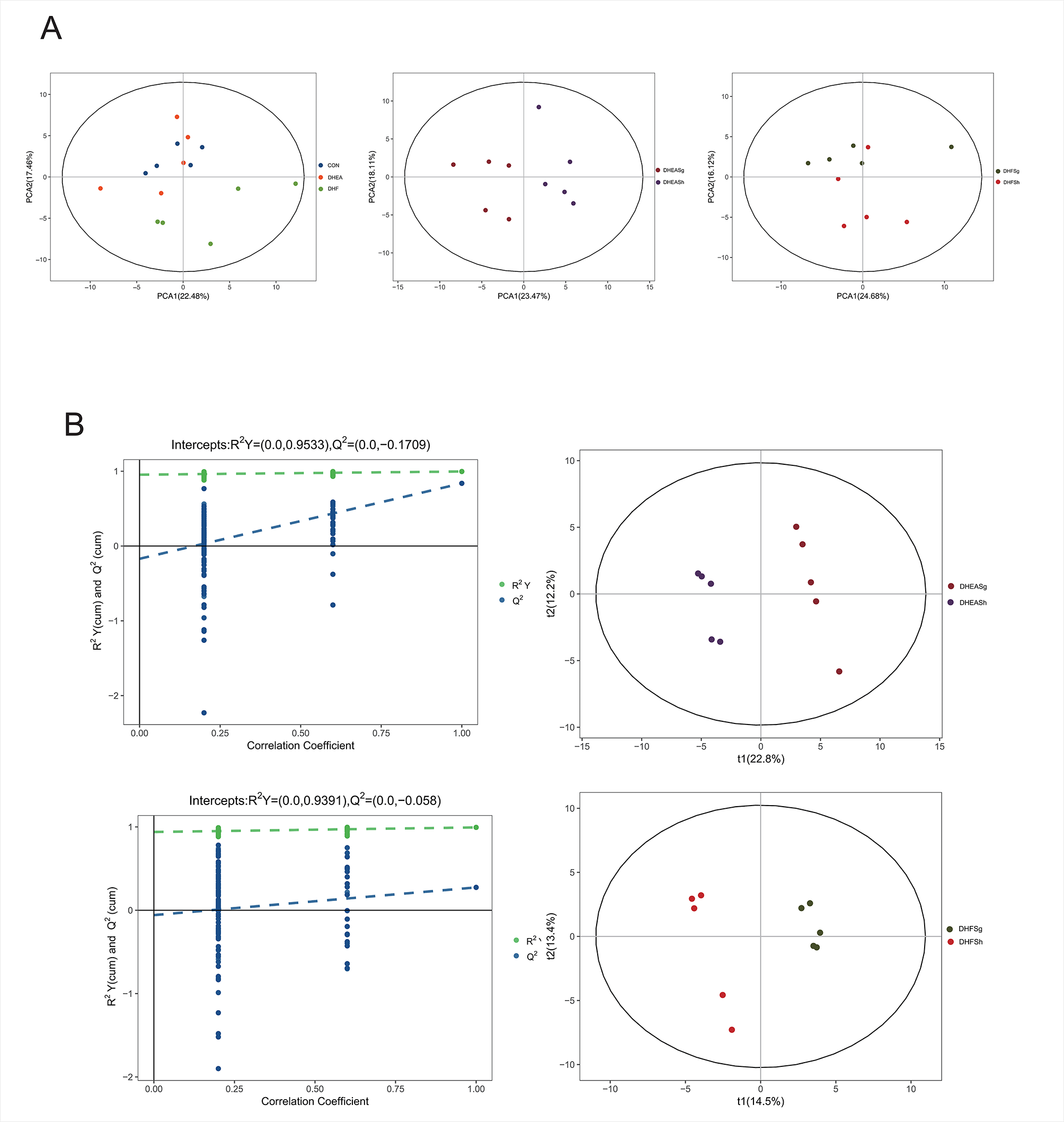

Supplement: Supplementary Figure 2 — Effects of sleeve gastrectomy (SG) on the alterations in gut metabolite composition. (A) Principal coordinates analysis of fecal metabolites. (A) CON, DHEA, and DHF; (B) DHEAsg and DHEASh; (C) DHFSg and DHFSh. (B) OPLS-DA analysis. (A) permutations in DHEASg and DHEAsh; (B) OPLS-DA plot between DHEASg and DHEAsh groups; (C) permutations in DHFSg and DHFSh; d OPLS-DA plot between DHFSg and DHFSh groups. [file Image_2.tif]

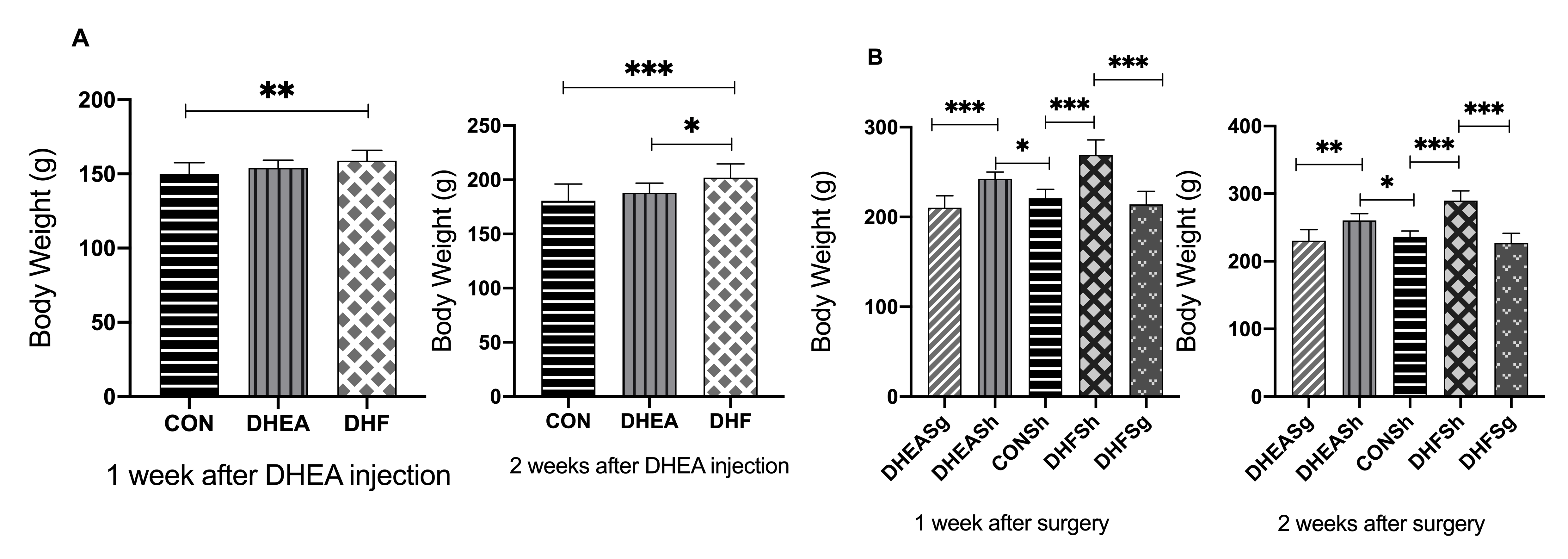

Supplement: Supplementary Figure 3 — Changes in body weight before (A) and after (B) surgery. *p < 0.05. **p < 0.01. ***p < 0.001. [file Image_3.tiff]
