# Supplementary material for: Effects of Sleeve Gastrectomy on Fecal Gut Microbiota and Short-Chain Fatty Acid Content in a Rat Model of Polycystic Ovary Syndrome
Source: Front Endocrinol (Lausanne). 2021 Nov 11;12:747888. doi: 10.3389/fendo.2021.747888 (PMC8631770; doi:10.3389/fendo.2021.747888)
Supplement: Supplementary Information — Supplementary methods (.doc). [file Table_1.docx]

***Supplementary Information***

1. Animals and diet

All experimental procedures were approved by the Committee on Animal Experiments of the Fujian Provincial Hospital (Fujian, China), according to the national legislation for animal care.

1. Surgical procedure

For SG, the procedure was as follows. A 4 cm incision was made 1 cm below the xiphisternum. The stomach was carefully exposed and isolated from the abdominal cavity. All vessels around the greater curvature were ligated using 6-0 silk suture. Two vessel forceps were placed along the greater curvature of the stomach, from the antrum to the fundus, across the proventriculus. The fundus of the stomach and most of the gastric body (70% of the total stomach) were removed. The remaining stomach was sutured using 5-0 silk suture one single layer by one. For sham surgery, all surgical incisions and the isolation of the peri-gastric tissue and blood vessels were in line with those performed for SG. After isolation, a 1 cm incision was made at the connection of the anterior stomach and glandular stomach on the side of the great bend of the stomach, and then the gastric tissue was sutured in situ.

1. Sequencing and analysis of gut microbiota

The PCR amplicon was analyzed and separated on a 2% agarose gel, which was purified using the AxyPrep DNA Gel Extraction Kit (Axygen Biosciences, Union City, CA, USA) according to the manufacturer’s instructions, and quantified using Qubit®2.0 (Invitrogen, Carlsbad, CA, USA). Afterwards, a library was established and sequenced using Illumina MiSeq PE300 (Illumina, Inc., CA, USA).

After assembly, quality filtering as well as random extraction of sequences at 97% similarity were conducted, and the operational taxonomic units for the species category were procured.

1. Fecal SCFAs

Target metabolites were dissolved in methanol, ultrapure water, sodium hydroxide solution, or hydrochloric acid solution at a concentration of 5 mg/mL. Depending on the solvent, all mixtures containing the target metabolites were mixed with a storage calibration solution.

Samples were thawed on an ice bath to avoid sample degradation. Approximately 10 mg of cecal samples was transferred to a 1.5 mL centrifuge tube and ground with zirconia beads by adding 25 μL of water for 3 min. Then, 185 μL of acetonitrile:methanol (8:2) was added and centrifuged at 18000 rpm and 4 °C for 20 min to extract the metabolites. The supernatant was transferred to a 96-well plate. Twenty microliters of methyl chloroformate(MCF) were added to each well, derivatized at 30 °C for 60 min, diluted with ice-cold methanol, refrigerated at –20 °C for 20 min, and centrifuged at 4000 ×*g* and 4 °C for 30 min. The supernatant (135 μL) and internal standard (15 μL) were mixed into a new 96-well plate.

Ultra-performance liquid chromatography-tandem mass spectrometry (ACQUITY UPLC-Xevo TQ-S, Waters Corp., Milford, MA, USA), which was conducted on an instrument equipped with electrospray ionization (ESI) source, was performed to quantify the metabolites. This system was a binary gradient elution system, which consisted of acetonitrile containing 0.1% formic acid (A) and 0.1% aqueous formic acid solution containing 1% acetonitrile (B). The gradient program was as follows: 0–1 min 5% B, 1–12 min 5%–80% B, 12–15 min 80%–95% B, 15–16 min 95%–100% B, 16–18 min 100% B, 18–18.1 min 100%–5% B, 18.1–20 min 5% B. The flow rate was 0.4 mL/min. A 5 μL aliquot of each sample was run on a ACQUITY UPLC® BEH C18 capillary column (2.1 × 100 mm ID, 1.7 μm, Waters Corp.). The programed temperature of the column was maintained at 40 °C. The mass spectrometry system was individually detected in ESI positive (ESI+) and ESI negative (ESI–) ion models. The optimized conditions were as follows: source temperature at 150 °C, desolvation gas flow at 1000 L/h at 550 °C.

Principal component analysis (PCA) and orthogonal partial least squares discriminant analysis (OPLS-DA) were conducted to visualize the quality of the metabolic profiling and metabolic alterations between groups. Significantly shifted metabolites with variable importance in projection values >1 in the OPLS-DA model, as same as differing p-values determined using the Student’s *t*-test (p < 0.05), were selected in SG and their corresponding sham-operated group. PCA and OPLS-DA were performed using Metaboanalyst 4.0.
